# Monitoring Adolescent and Young Adult Patients With Cancer via a Smart T-Shirt: Prospective, Single-Cohort, Mixed Methods Feasibility Study (OncoSmartShirt Study)

**DOI:** 10.2196/50620

**Published:** 2024-05-01

**Authors:** Emma Balch Steen-Olsen, Helle Pappot, Maiken Hjerming, Signe Hanghoej, Cecilie Holländer-Mieritz

**Affiliations:** 1 Department of Oncology, Centre for Cancer and Organ Diseases, Copenhagen University Hospital - Rigshospitalet, Copenhagen, Denmark; 2 Department of Clinical Medicine, Faculty of Health and Medical Sciences, University of Copenhagen, Copenhagen, Denmark; 3 Department of Oncology, Zealand University Hospital, Naestved, Denmark

**Keywords:** smart T-shirt, AYA, oncology, home monitoring, patients' perspective, perspective, perspectives, experiences, experience, youth, adolescent, adolescents, smart, monitoring, biometric, sensor, sensors, young adult, young adults, feasibility, cancer, cancers, electrode, electrodes, adherence, mobile phone

## Abstract

**Background:**

Wearables that measure vital parameters can be potential tools for monitoring patients at home during cancer treatment. One type of wearable is a smart T-shirt with embedded sensors. Initially, smart T-shirts were designed to aid athletes in their performance analyses. Recently however, researchers have been investigating the use of smart T-shirts as supportive tools in health care. In general, the knowledge on the use of wearables for symptom monitoring during cancer treatment is limited, and consensus and awareness about compliance or adherence are lacking.

**Objectives:**

The aim of this study was to evaluate adherence to and experiences with using a smart T-shirt for the home monitoring of biometric sensor data among adolescent and young adult patients undergoing cancer treatment during a 2-week period.

**Methods:**

This study was a prospective, single-cohort, mixed methods feasibility study. The inclusion criteria were patients aged 18 to 39 years and those who were receiving treatment at Copenhagen University Hospital - Rigshospitalet, Denmark. Consenting patients were asked to wear the Chronolife smart T-shirt for a period of 2 weeks. The smart T-shirt had multiple sensors and electrodes, which engendered the following six measurements: electrocardiogram (ECG) measurements, thoracic respiration, abdominal respiration, thoracic impedance, physical activity (steps), and skin temperature. The primary end point was adherence, which was defined as a wear time of >8 hours per day. The patient experience was investigated via individual, semistructured telephone interviews and a paper questionnaire.

**Results:**

A total of 10 patients were included. The number of days with wear times of >8 hours during the study period (14 d) varied from 0 to 6 (mean 2 d). Further, 3 patients had a mean wear time of >8 hours during each of their days with data registration. The number of days with any data registration ranged from 0 to 10 (mean 6.4 d). The thematic analysis of interviews pointed to the following three main themes: (1) the smart T-shirt is cool but does not fit patients with cancer, (2) the technology limits the use of the smart T-shirt, and (3) the monitoring of data increases the feeling of safety. Results from the questionnaire showed that the patients generally had confidence in the device.

**Conclusions:**

Although the primary end point was not reached, the patients’ experiences with using the smart T-shirt resulted in the knowledge that patients acknowledged the need for new technologies that improve supportive cancer care. The patients were positive when asked to wear the smart T-shirt. However, technical and practical challenges in using the device resulted in low adherence. Although wearables might have potential for home monitoring, the present technology is immature for clinical use.

## Introduction

Patients with cancer can be exposed to several treatments (eg, surgery, radiation, chemotherapy, and hormone therapy), individually or in combination, depending on their disease and stage. Cancer treatment is known to cause acute side effects [1-3]. The degree of symptoms and side effects depends on the type of cancer, the treatment modality, and the pre-existing comorbidity [1,4,5]. During oncological treatment, the patients may need acute hospitalization due to side effects, while in other cases, side effects are related to poor treatment compliance and reduced quality of life (QoL) [6].

Several studies have emphasized that patients and health care professionals can assess and perceive symptoms and side effects differently [7,8]. This is exemplified by the fact that health care professionals tend to underestimate patients’ symptoms [9]. The development of side effects and symptoms often results in a deterioration of the patient’s health condition, affecting the patient’s QoL [10,11]. This applies especially to adolescents and young adults (AYAs) with cancer [12].

There is an increased focus on home monitoring to help patients manage their symptoms and side effects. Patient-generated health data can provide health care professionals with valuable information. One type of patient-generated health data is biometric sensor data, which are typically collected by wearables [13-15]. A wearable device is a noninvasive wireless sensor that monitors and collects health parameters [13,16]. A newer type of wearable is a smart T-shirt with biometric sensors embedded in the fabric. Wearables allow health professionals to monitor an increased number of health parameters on various biometric data points.

The data collected from wearables are predicted to be exact and comparable to data collected from conventional medical measuring devices [17-19]. These new technologies allow for the more extensive passive monitoring of patients in their home environment and may minimize the burden resulting from hospital visits [6,16,20-23]. In addition, wearables ensure exact information without recall and reporting bias, which hopefully results in better cancer treatment [20,24-26]. However, studies that investigate the use of wearables in an oncological setting are limited [24,27-29]. Furthermore, it has been stated that there is a lack of consensus and awareness about compliance with or adherence to wearables. These are essential parts of using and comparing collected biometric sensor data [30].

Many existing and new technologies are not developed or evaluated based on users’ perspectives and sometimes do not adequately meet the needs of their target groups [31-33]. The AYA patient group frequently uses new technologies, such as wearables [34]. AYAs thus have unique and beneficial knowledge, which is why patients’ involvement in the study design and in feasibility assessment can be extremely useful [35].

The aim of this study was to evaluate feasibility based on adherence to and experiences with using a smart T-shirt for the remote monitoring of biometric sensor data among AYA patients undergoing cancer treatment during a 2-week test period.

## Methods

### Ethical Considerations

This study was an investigator-driven partnership between the Department of Oncology, Rigshospitalet, and Chronolife and was registered at ClinicalTrials.gov (trial number: NCT05235594). This study conformed to the General Data Protection Regulation guidelines and was registered at the Capital Region of Denmark (registration number: P-2021-357). The trial was approved by the local division for IT and Medico Technology in the Capital Region of Denmark and was a collaboration between the Department of Oncology, Rigshospitalet; the Department of Innovation, Rigshospitalet; and the Telemedical Knowledge Center, Capital Region of Denmark. Approval from the National Committee on Health Research Ethics was not required for this trial in the Danish context when this study was conducted. Informed consent was obtained from all patients involved in this study. The patients received verbal and written information. Written informed consent was required, and patients were informed that it was possible to withdraw from this study at any time during the study period. No financial compensation was provided.

### Study Design

The OncoSmartShirt study was a prospective, single-cohort, mixed methods study that investigated the feasibility of using the Chronolife smart T-shirt (Keesense) during cancer treatment. This smart T-shirt was designed with multiple fully embedded sensors and electrodes, which engendered 6 different measurement flows continuously [25]. Before this study was conducted, the project was presented to a group of AYAs with cancer at a social meeting with the “Kræftværket” cancer network group. The participants provided the researchers with their inputs and perspectives on the study design to make it relevant and feasible. The acceptance and comfort of wearing the Chronolife smart T-shirt throughout the day (8 h/d) for 2 weeks (14 d) were investigated among all enrolled patients.

The inclusion criteria were young patients with cancer aged 18 to 39 years (defined as *AYAs*) and those who were receiving antineoplastic treatment at the Department of Oncology and Department of Haematology of the Centre for Cancer and Organ Diseases, Copenhagen University Hospital - Rigshospitalet, Copenhagen, Denmark. Other inclusion criteria were having the ability to read and speak Danish and having no serious cognitive deficits. There were no requirements regarding specific cancer diagnoses, and both patients in curative care and patients in palliative care could be included. Inclusion in this study did not interfere with the planned oncological treatment.

Further details on the OncoSmartShirt study can be reviewed in the previously published protocol article [36]. This paper reports results from 10 patients with cancer aged under 39 years (defined as *AYAs*). The decision to have a sample size of 10 AYA patients was influenced by the feasibility study design, which does not require a formal power calculation but aligns with the sample sizes used for similar studies in the literature [36]. In the previously published protocol, the plan was to also include 10 patients with cancer older than 65 years (defined as *elderly*). However, due to the results from the AYA cohort, the research group omitted the inclusion of the second cohort of older patients.

### Device

**Figure 1.**
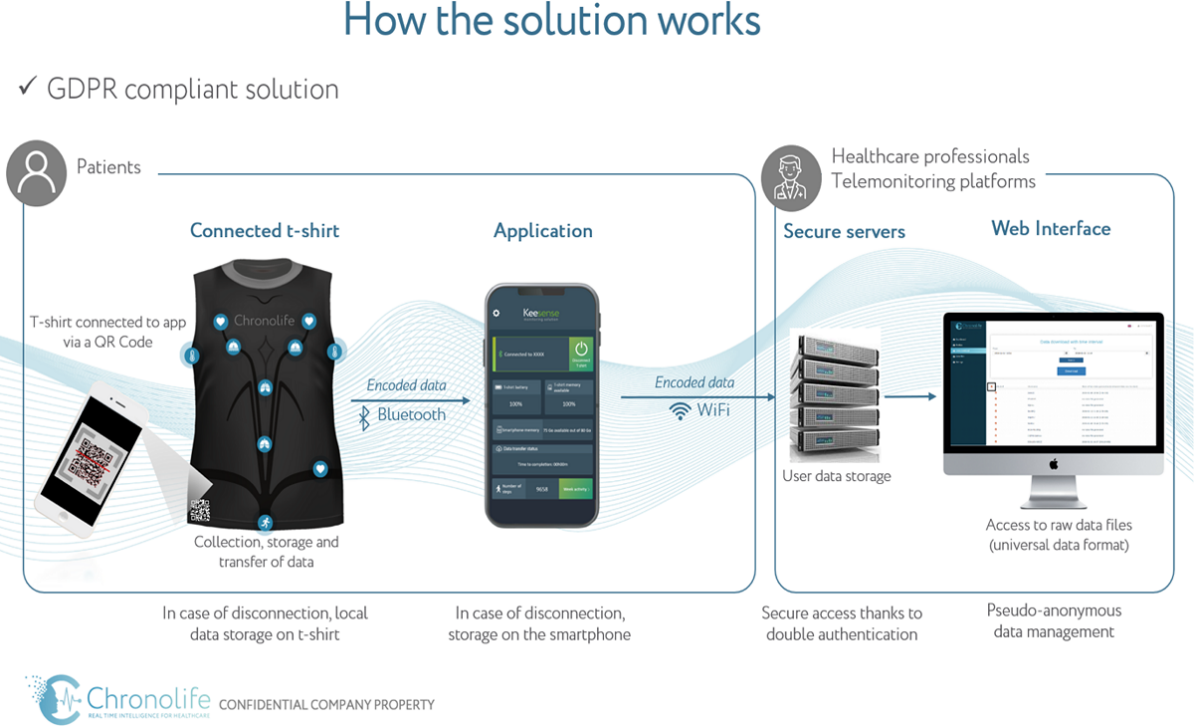
Framework for the OncoSmartShirt study (previously published figure from our protocol article [36]). GDPR: General Data Protection Regulation.

The device in this study consisted of the following four units: a washable smart T-shirt from Chronolife; a companion smartphone app; a secure, accredited data hosting server; and a web interface (Figure 1) [37]. The Chronolife smart T-shirt was designed for everyday use. It had electrical sensors embedded, allowing for the detection of the following six physiological parameters: electrocardiogram (ECG) measurements (beats/min), thoracic respiration (respirations/min), abdominal respiration (respirations/min), thoracic impedance (kΩ), physical activity (steps), and skin temperature (°C) [37]. A rechargeable battery powered the sensors. Additionally, a memory card that stores data and a Bluetooth interface that transmits data were fully integrated into the smart T-shirt and sealed in water-resistant coatings. The smart T-shirt was commercialized and Conformité Européenne–marked for the consumer market.

The smart T-shirt connected to the smartphone app via a QR code located on the smart T-shirt. Bluetooth Low Energy transmitted collected health data to the connected smartphone app designed for storage. The app transmitted data to a data hosting server that stored and provided data for a web interface, which was used for analysis and algorithm training [37].

The devices—the smart T-shirt and connected smartphone—were supplied by the hospital and returned after study termination. For data safety reasons, the patients could not use their own smartphones. Each smart T-shirt was produced based on individual patients’ size measurements. No biometric data on health parameters collected by the smart T-shirt were sent to health care professionals during this study. The patients could contact a study assistant if they experienced technical difficulties, but this was not a requirement.

### Patient Feedback

Immediately after the intervention period, qualitative individual telephone interviews were performed with the participants, allowing them to elaborate on their experiences with using the smart T-shirt. A semistructured interview guide was used. The interview guide was developed by SH based on research in the field [38,39] and consisted of questions about the pros and cons of using the smart T-shirt, material and appearance, identity and social stigma, behavioral changes, and ethics [36]. The interviews were performed by SH, who did not have prior knowledge about the participants and vice visa.

Patients were also asked to complete a quantitative paper questionnaire (handed out by a study assistant) concerning their experiences with wearing the smart T-shirt. The questionnaire consisted of 21 items, of which 14 were statements; patients could use a 5-point scale to indicate whether they agreed or disagreed with the statements. The remaining items consisted of free-text questions and items for gathering data on patients’ estimates of wear time. The patients were asked to complete the questionnaire once. The questionnaire was prepared by Chronolife.

### Variables

The primary end point was to assess the feasibility of using the Chronolife smart T-shirt based on adherence, which was defined as the number of included patients who used the smart T-shirt (wear time) at least 8 hours per day during the 2-week period. The wear times were obtained from the processed data provided by Chronolife. Wear time was not self-reported. Secondary end points were patient feedback, which was obtained as described in the *Patient Feedback* section, and technical feasibility in a Danish health care system, including data quality. Explorative end points were changes in the health parameters monitored.

### Data Analysis

Chronolife conducted the analysis of the collected health data. Qualitative data from the telephone interviews were transcribed and analyzed thematically, according to Braun and Clarke’s [40] approach. Text coding involved reading and rereading the transcriptions to identify and categorize concepts across data. Concepts relevant to the research question were highlighted using colored marks in the transcriptions and then sorted into themes. One researcher did the coding (SH), which was discussed thoroughly with a senior researcher (HP) until a consensus was reached.

## Results

### Baseline Demographics and Characteristics

The recruitment of patients for the project was organized by using a Facebook post (Meta Platforms Inc; May 2021) about the project, which was posted in a closed group for young patients with cancer. The patients thus contacted the research groups if they wanted to participate in the project. A total of 10 patients (female: n=5; male: n=5) aged 22 to 30 (median 27, IQR 24.5-29.5) years were included during the inclusion period (March to June 2022). The types of cancer were leukemia (n=3), lymphoma (n=2), breast cancer (n=2), central nervous system cancer (n=1), testis cancer (n=1), and malignant melanoma (n=1).

### Feasibility and Data Quality

As shown in Table 1, the number of days with wear times of >8 hours during the study period varied from 0 to 6 (mean 2 d). Only 3 patients had a mean wear time of >8 hours during each of their days with data registration. The number of days with any data registration varied from 0 to 10 (mean 6.4 d). No one managed to wear the smart T-shirt 8 hours per day for 14 days straight. Further, 4 patients had no data registrations at all. For 3 of these patients, the connection between the smart T-shirt and the smartphone app was unsuccessful because of technical issues or malfunctions, and for the last patient, the connection was successful, but the patient did not use the smart T-shirt due to disease-related issues.

**Table 1.** Wear time data for the smart T-shirt.

	Number of sessions^a^	Number of days with data registration	Number of days with a wear time of >8 h	Maximum wear time per session (h)	Wear time per day (h), mean^b^	Total wear time (h)	Data quality (%), mean^b^
Patient 1	20	10	6	19.1	10.4	110.4	63
Patient 2	—^c^	—	—	—	—	—	—
Patient 3	—	—	—	—	—	—	—
Patient 4	0	0	0	0	0	0	—
Patient 5	—	—	—	—	—	—	—
Patient 6	2	2	0	0.08	0.065	0.13	92
Patient 7	15	9	4	14.4	8	76	90
Patient 8	12	10	2	20	4.2	34	87
Patient 9	16	9	3	14.4	10.4	93.8	65
Patient 10	5	5	1	8.6	4.1	20.4	63

a The number of sessions is the total number of times the smart T-shirt was worn.

b SDs were not available from Chronolife.

c Not available.

The heart rate was calculated based on an ECG segment that was considered reliable by the manufacturer’s data cleaning algorithm. The data quality was defined as the following ratio: the length of the session with heart rate values available divided by the total length of the session. The mean data quality for patients from whom data were collected varied from 63% to 92% (mean 77%). In this study, the data quality value was based on the quality of the heart rate values. The data quality can vary with multiple factors. The most important factor was the fit of the smart T-shirt; if the smart T-shirt was too large, the electrodes for the ECG would not have optimal contact with the skin, which would cause noise and artifacts in the collected data.

As variable compliance and data quality were noted, the analysis of the collected health data has been omitted due to the risk of the misinterpretation of the results.

### Feedback From Patients

#### Interviews

##### Overview of Thematic Analysis

The thematic analysis was based on telephone interviews. The interviews lasted between 8 and 21 (mean 12) minutes. The thematic analysis pointed to the following three main themes: (1) the smart T-shirt is cool but does not fit patients with cancer, (2) the technology limits the use of the smart T-shirt, and (3) the monitoring of data increases the feeling of safety.

##### Theme 1: The Smart T-Shirt Is Cool but Does Not Fit Patients With Cancer

The smart T-shirt was described as “soft,” and some participants did not even notice wearing it. All participants agreed that the smart T-shirt had a nice design, which they did not associate with anything patient-like. The term “cool” reflects the look of the smart T-shirt rather than the sensation experienced while wearing it. There were different opinions about how the body’s temperature was affected by the smart T-shirt. A few participants got extremely hot and could not bear to wear it for a long time, and this worsened when these participants were physically active. However, others thought it felt cool in terms of body temperature. The smart T-shirt was closed with a zipper at the side. Most participants had a hard time zipping it by themselves. Some described the smart T-shirt as a bit too long, and several were bothered by the transverse bands on the smart T-shirt that contained the measuring equipment, which was not elastic like the rest of the smart T-shirt. For the sake of measurements, the smart T-shirt was designed to fit very tightly, which was a problem for several participants who had undergone surgery or were experiencing medical side effects. Moreover, the women could not wear a bra under the smart T-shirt. The consequence of all of these challenges was that it was too difficult for the participants to wear the smart T-shirt as prescribed.

##### Theme 2: The Technology Limits the Use of the Smart T-Shirt

The participants generally had challenges with getting the technology in the smart T-shirt and smartphone to work. Several participants described problems with charging the smart T-shirt. The smartphone’s connection to Wi-Fi was associated with some problems. Additionally, 1 participant noted that the app stopped working one night when his smartphone made automatic updates. In general, the participants requested the smart T-shirt to connect to their own smartphones, so that they did not have to carry 2 smartphones simultaneously. The technical challenges were experienced as barriers to wearing the smart T-shirt. One of the participants explained that as a patient with cancer, they had very little energy to overcome everyday things (eg, technology that does not work).

##### Theme 3: The Monitoring of Data Increases the Feeling of Safety

All participants described the smart T-shirt as a useful and important invention that met their need for safety when being released from the hospital and not being monitored by health professionals anymore. Most participants believed that the smart T-shirt could be used optimally if the health professionals could track their health parameters from the hospital and then contact them if something looked abnormal (eg, in cases of changes in heart rate or breathing). One of the participants expressed that the monitoring was like “bringing the hospital home” because it provided him with the same feeling of safety as when they were hospitalized. Another explained that he felt less ill when he was at home and that the smart T-shirt could play a key role in getting home under safe conditions. However, there was also a participant who explained that constant monitoring from the hospital would require the patients to be introduced to their tracking data, so that they would know what was normal and what was not. Some participants explained that being released from the hospital could be very concerning, especially because of their increased focus on their body and whether it behaved differently. In general, the participants strongly desired to follow bodily signs during cancer treatment. One participant believed that it would have been more motivating to use the smart T-shirt if he could follow and view the collected data on the smartphone.

### Quantitative Questionnaire

Of the 10 patients, 8 responded to the quantitative questionnaire, providing 165 out of 210 (item response rate: 78.6%) possible answers. The patients’ answers to the 14 statements in the questionnaire are illustrated in Table 2. The answers were very different among the patients, but in general, the patients had confidence in the product and believed that their physician could use the collected health data. In addition, several patients were concerned about whether the smart T-shirt worked correctly and whether it would limit their daily activities.

**Table 2.** The patients’ responses to the questionnaire concerning their experiences with the smart T-shirt (Keesense). The questionnaire was prepared by the smart T-shirt manufacturer (Chronolife).

Questionnaire statements	Patients’ responses, n					Total responses, N
	1 (strongly disagree)	2 (disagree)	3 (neutral)	4 (agree)	5 (strongly agree)	
“Before starting the project, I had no fears regarding the medical device - the smart t-shirt and the phone”	0	0	0	1	7	8
“I think using this device will help my doctors monitor my condition more closely”	0	0	0	4	4	8
“I am afraid that using the Keesense medical device may affect my daily activities”	1	4	1	1	1	8
“I think using the Keesense medical device can help me be more active”	0	4	3	0	1	8
“I am concerned about a possible malfunction of the Keesense medical device”	3	2	1	2	0	8
“After being shown and taught how to use the Keesense medical device, I was confident that I could then use it”	0	0	1	4	3	8
“I think the smart t-shirt was easy to use”	0	1	2	4	1	8
“I felt comfortable with the use of the Keesense medical device”	0	3	1	3	1	8
“I easily forget that I am wearing the smart t-shirt”	1	2	2	1	2	8
“I can accomplish my daily activities with the smart t-shirt”	0	0	3	1	4	8
“I can easily do physical activity with the smart t-shirt”	0	1	4	1	2	8
“I find the Keesense medical device easy to use”	0	2	2	2	1	7^a^
“I sweat abnormally while wearing the Keesense smart t-shirt”	0	2	4	0	1	7^a^
“I have skin itching and/or irritation”	4	2	0	1	0	7^a^

a One patient did not provide an answer for the statement.

## Discussion

### Principal Findings

The OncoSmartShirt study was a feasibility study that tested a smart T-shirt for the home monitoring of AYA patients with cancer from a public health care hospital in Denmark. To investigate adherence, we had predefined a preferred wear time of 8 hours daily for 2 weeks [36]. Unfortunately, none of the included patients achieved this. This finding is similar to the results from a comparable study conducted by Höllander-Mieritz et al [41] that investigated adherence to a smartwatch during radiotherapy among patients with head and neck cancer. Specific literature reviews show that it is possible to achieve high adherence to wearable technology in an oncology setting [30,42]. In general however, there is a tendency for compliance to decrease as the length of the study period increases. In addition, the exact desired wear time per day is not specified in several studies. This can contribute to the fact that it can be difficult to compare adherence across different studies.

We included qualitative and quantitative data in this study, and even though the wear time target was not met, this feasibility study resulted in knowledge about patients’ experiences with the smart T-shirt and why the patients did not use the smart T-shirt in the predetermined time. In the qualitative interviews, we identified a discrepancy between the need for the smart T-shirt and the design of the smart T-shirt. The T-shirt met the patient’s needs in terms of monitoring their health, but at the same time, it was not designed for patients with cancer experiencing treatment-induced side effects, such as gastrointestinal problems, increased body heat, and scars.

Another explanation for the lack of compliance is the technical challenges that some of the participants encountered. Prior to the completion of this study, we assumed that the group of AYAs would have greater technical ability and thus experience fewer technical problems when compared to older patients with cancer [43]. Based on the questionnaire, it appeared that most participants were confident with the technology and could use the smart T-shirt and the smartphone. However, in reality, 3 participants never connected their smart T-shirts to the smartphone app correctly, and several others experienced technical issues. In addition, it emerged from the interviews that patients with cancer do not have the energy to deal with technical problems associated with, for example, a smart T-shirt.

Responses to the questionnaire varied widely among participants and spanned the entire scale. Nevertheless, it is important to emphasize that the smart T-shirt manufacturer (Chronolife) prepared the questionnaire, which probably increased the risk of a ceiling effect among the answers [44].

To our knowledge, no studies have investigated the use of a smart T-shirt in an oncology setting, but there have been a few studies in the field of cardiology that primarily investigated if the monitoring of ECGs via a smart T-shirt can replace Holter monitoring [17,18,45]. In general, compliance is better in cardiology studies, but unlike the participants in the OncoSmartShirt study, the participants in those aforementioned cardiology studies were healthy and were not undergoing treatment. Thus, healthy participants in cardiology studies probably do not have the same challenges and annoyances as those among AYA patients with cancer [17,18]. Further, because of the low adherence and the patient experiences identified in patient interviews and the quantitative questionnaire, the research group excluded the preplanned group of older patients from this study. We believed that compared to the AYA patients included in this study, older participants would have experienced the same amount of issues (if not more) with the smart T-shirt and the setup with an extra phone. Therefore, we did not find it ethical to proceed with their inclusion in this study. However, we believe that age would not be an issue with a less demanding technical setup.

This study highlights the importance of investigating practical and technical feasibility. Practical feasibility refers to the specific wearable chosen, including the design, comfort, number of connected devices, and need for charging and maintenance. The smart T-shirt was not comfortable for our patient population. The patients had to have an extra phone to secure the setup and ensure safe data transfer. Both the smart T-shirt and the phone required charging, and the smart T-shirt also needed to be washed, which added to the patients’ tasks. Technical feasibility refers to the setup for securing the data transfer, data quality, and reliable data. In this study, the data were safely transferred, but the data quality fluctuated.

For future studies investigating wearables, we suggest that the device be simple, comfortable, and minimally disturbing for the patient. The technical setup must also be simple; a possibility could be using the bring-your-own-device study design if the data can be securely transferred and if the data quality is sufficient. However, caution is advised for conducting bring-your-own-device studies in research due to the potential imbalance [46]. We also recommend having a technical backup team that patients can contact and providing resources for electronic health education to patients and health care professionals. Finally, studies must assist in determining the potential uses of the data collected.

Although wearables might have the potential to be used in selected patient groups who need monitoring for a period of time, it is essential that the wearables can be worn and accepted by the patients and that the technical setup is as convenient as possible.

### Conclusion

The OncoSmartShirt study was a feasibility study that investigated the use of a Chronolife smart T-shirt for the home monitoring of vital parameters among AYA patients with cancer during treatment. This study showed that AYA patients with cancer could not wear a smart T-shirt 8 hours per day for 2 weeks. However, this study revealed new and important perspectives and knowledge, which, among other things, pointed to why it can be challenging to achieve high compliance in this type of study. Furthermore, this study, as well as the patients, emphasized that wearables have potential. However, this area requires more research to develop the proper setup with minimal effort on the part of patients. Hopefully, in the long term, wearables can help improve the QoL for patients with cancer.

## Abbreviations

AYA: adolescent and young adult
ECG: electrocardiogram
QoL: quality of life
